# Cardiometabolic Morbidity (Obesity and Hypertension) in PTSD: A Preliminary Investigation of the Validity of Two Structures of the Impact of Event Scale-Revised

**DOI:** 10.3390/jcm13206045

**Published:** 2024-10-10

**Authors:** Amira Mohammed Ali, Saeed A. Al-Dossary, Carlos Laranjeira, Maha Atout, Haitham Khatatbeh, Abeer Selim, Abdulmajeed A. Alkhamees, Musheer A. Aljaberi, Annamária Pakai, Tariq Al-Dwaikat

**Affiliations:** 1 Department of Psychiatric Nursing and Mental Health, Faculty of Nursing, Alexandria University, Smouha, Alexandria 21527, Egypt; amira.mohali@alexu.edu.eg; 2 Department of Psychology, College of Education, University of Ha’il, Ha’il 1818, Saudi Arabia; s.aldossary@uoh.edu.sa; 3 School of Health Sciences, Polytechnic University of Leiria, Campus 2, Morro do Lena, Alto do Vieiro, Apartado 4137, 2411-901 Leiria, Portugal; carlos.laranjeira@ipleiria.pt; 4 Centre for Innovative Care and Health Technology (ciTechCare), Polytechnic University of Leiria, Campus 5, Rua das Olhalvas, 2414-016 Leiria, Portugal; 5 Comprehensive Health Research Centre (CHRC), University of Évora, 7000-801 Évora, Portugal; 6 School of Nursing, Philadelphia University, Amman 19392, Jordan; m.atout@philadelphia.edu.jo; 7 Department of Clinical Nursing, Faculty of Nursing, Yarmouk University, Irbid 21163, Jordan; haitham.khatatbeh@yu.edu.jo; 8 Psychiatric and Mental Health Nursing Department, Faculty of Nursing, Mansoura University, Mansoura 35516, Egypt; abeer_ai_selim_again@yahoo.com; 9 College of Nursing, King Saud bin Abdulaziz University for Health Sciences, Riyadh 11481, Saudi Arabia; 10 King Abdullah International Medical Research Center, Riyadh 11481, Saudi Arabia; 11 Department of Psychiatry, College of Medicine, Qassim University, Buraidah 52571, Al Qassim, Saudi Arabia; 12 Department of Internal Medicine, Section Nursing Science, Erasmus University Medical Center (Erasmus MC), 3015 GD Rotterdam, The Netherlands; musheer.jaberi@gmail.com; 13 Institute of Nursing Sciences, Basic Health Sciences and Health Visiting, Faculty of Health Sciences, University of Pécs, 7621 Pécs, Hungary; annamaria.pakai@etk.pte.hu; 14 Community and Mental Health Nursing Department, Faculty of Nursing, Jordan University of Science and Technology, Irbid 22110, Jordan; tnaldwaikat@just.edu.jo

**Keywords:** impact of event scale-revised/IES-R/posttraumatic stress disorder/PTSD, cutoff score/cutoff point, three factors/six factors/dimensions, receiver operator curve (ROC), cardiovascular disorders/CVDs/cardiometabolic*/hypertension, diet*/high-sugar/high fat, obesity/high body mass index/metabolic dysfunction, sleep disturbance/irritability/emotional numbing, smoking/behavioral risk factors, dental healthcare workers

## Abstract

**Background:** Posttraumatic stress disorder (PTSD) and/or specific PTSD symptoms may evoke maladaptive behaviors (e.g., compulsive buying, disordered eating, and an unhealthy lifestyle), resulting in adverse cardiometabolic events (e.g., hypertension and obesity), which may implicate the treatment of this complex condition. The diagnostic criteria for PTSD have lately expanded beyond the three common symptoms (intrusion, avoidance, and hyperarousal). Including additional symptoms such as emotional numbing, sleep disturbance, and irritability strengthens the representation of the Impact of Event Scale-Revised (IES-R), suggesting that models with four, five, or six dimensions better capture its structure compared to the original three-dimensional model. **Methods:** Using a convenience sample of 58 Russian dental healthcare workers (HCWs: mean age = 44.1 ± 12.2 years, 82.8% females), this instrumental study examined the convergent, concurrent, and criterion validity of two IES-R structures: IES-R3 and IES-R6. **Results:** Exploratory factor analysis uncovered five factors, which explained 76.0% of the variance in the IES-R. Subscales of the IES-R3 and the IES-R6 expressed good internal consistency (coefficient alpha range = 0.69–0.88), high convergent validity (item total correlations r range = 0.39–0.81, and correlations with the IES-R’s total score r range = 0.62–0.92), excellent concurrent validity through strong correlations with the PTSD Symptom Scale-Self Report (PSS-SR: r range = 0.42–0.69), while their criterion validity was indicated by moderate-to-low correlations with high body mass index (BMI: r range = 0.12–0.39) and the diagnosis of hypertension (r range = 0.12–0.30). In the receiver-operating characteristic (ROC) curve analysis, all IES-R models were perfectly associated with the PSS-SR (all areas under the curve (AUCs) > 0.9, *p* values < 0.001). The IES-R, both hyperarousal subscales, and the IES-R3 intrusion subscale were significantly associated with high BMI. Both avoidance subscales and the IES-R3 intrusion subscale, not the IES-R, were significantly associated with hypertension. In the two-step cluster analysis, five sets of all trauma variables (IES-R3/IES-R6, PSS-SR) classified the participants into two clusters according to their BMI (normal weight/low BMI vs. overweight/obese). Meanwhile, only the IES-R, PSS-SR, and IES-R3 dimensions successfully classified participants as having either normal blood pressure or hypertension. Participants in the overweight/obese and hypertensive clusters displayed considerably higher levels of most trauma symptoms. Input variables with the highest predictor importance in the cluster analysis were those variables expressing significant associations in correlations and ROC analyses. However, neither IES-R3 nor IES-R6 contributed to BMI or hypertension either directly or indirectly in the path analysis. Meanwhile, age significantly predicted both health conditions and current smoking. Irritability and numbing were the only IES-R dimensions that significantly contributed to current smoking. **Conclusions:** The findings emphasize the need for assessing the way through which various PTSD symptoms may implicate cardiometabolic dysfunctions and their risk factors (e.g., smoking and the intake of unhealthy foods) as well as the application of targeted dietary and exercise interventions to lower physical morbidity in PTSD patients. However, the internal and external validity of our tests may be questionable due to the low power of our sample size. Replicating the study in larger samples, which comprise different physical and mental conditions from heterogenous cultural contexts, is pivotal to validate the results (e.g., in specific groups, such as those with confirmed traumatic exposure and comorbid mood dysfunction).

## 1. Introduction

Posttraumatic stress disorder (PTSD) may develop in individuals witnessing direct or threatened exposure to traumatic events, abuse, and adversities, such as terrorist attacks, war/combat, rape, etc. [1,2,3]. The main PTSD symptoms include intrusion or rumination, avoidance, and hyperarousal. Individuals experiencing these symptoms frequently recover in the first month following trauma exposure. However, emotion regulation strategies that involve suppressing rather than modifying trauma-related emotional responses may account for the persistence of PTSD symptoms in certain groups (e.g., those unable to grieve) due to persistent activation of the hypothalamic–pituitary–adrenal (HPA) axis [4,5,6]. Stress susceptibility in PTSD is noted by increased activity of the anterior pituitary glucocorticoid receptor [7]. Parenthetically, cumulative research shows that PTSD is a systemic stress-related mental disorder, which evokes physiological, behavioral, and psychological responses that are conducive to the development of cardiovascular disorders (CVDs) [8,9]. 

Altered arousal and reactivity—a core component of the current Diagnostic and Statistical Manual of Mental Disorders (DSM)-5-TR diagnostic criteria of PTSD—is an acute sympathetic arousal in trauma response, which has been described during the Civil War as “soldiers’ heart” [10]. A possible causative effect of PTSD on CVDs has been reported in a longitudinal study following 320 normotensive individuals. Developing hypertension significantly correlated with six variables (age, educational level, body mass index (BMI), smoking, diabetes, and PTSD diagnosis). However, in regression analysis, only PTSD diagnosis was significantly associated with incident hypertension (multivariate hazard ratio = 1.94; 95% CI 1.11–3.40) [11]. In a case control study recruiting hypertensive and normotensive individuals from a city enduring war for 25 years—Bukavu in the Democratic Republic of Congo—hypertension was associated with greater exposure to man-made traumas (61 vs. 13%), PTSD (36 vs. 7%), major depressive disorder (MDD; 37 vs. 13%), and alcohol use disorder (23 vs. 4%, all *p* values < 0.001) [12]. Similarly, PTSD diagnosis and symptom severity in veterans were linked to a 29% increase in hypertension risk independent of MDD or depressive symptom severity, relative to veterans without lifetime diagnoses of either disease. However, MDD comorbidity in individuals with PTSD is associated with a considerably increased risk of hypertension (66%) [13].

The common interplay between PTSD and cardiometabolic dysregulations entails the accelerated activity of oxidative and inflammatory signaling due to biological/genetic and behavioral vulnerability in PTSD victims [8,14]. Genome-wide association and Mendelian randomization studies uncovered significant genetic correlations between PTSD and CVDs, with a causal link of PTSD to hypertension, but not the opposite. The correlations were stronger when summary statistics from CVDs and MDD were combined. The shared PTSD-CVDs risk incorporates genetic variants, which are involved in postsynaptic structure, synapse organization, and interleukin (IL)-7-mediated signaling pathways [14]. PTSD is also associated with several independent loci and single nucleotide polymorphisms in the PARK2 gene. Genetic variants associated with PTSD polygenic risk scores are suggestively associated with PTSD incidence and severity, as well as the incidence of metabolic syndrome (obesity, dyslipidemia, and insulin resistance) [15]. A robust positive genetic correlation links PTSD and metabolic syndrome, especially its obesity-related component, while longitudinal investigations indicate that PTSD may cause an increase in BMI, primarily in women [16]. This may justify the widespread prevalence of obesity among PTSD victims (odds ratio = 1.55, 95% CI: 1.32–1.82) [16,17,18,19,20].

Compared with PTSD-free individuals, PTSD patients display an increased behavioral risk for CVDs and obesity, including physical inactivity, smoking, and an unhealthy diet (intake of large amounts of soda, fast food, and energy from trans fatty acids, with a limited intake of fresh fruits and vegetables) [18,21]. Stress hormones and dysregulations of the limbic system and prefrontal cortex in PTSD individuals aggravate craving for carbohydrates and ultra-processed foods [22,23], evoking the development of eating disorders in a large proportion of PTSD patients [17,18]. Binge eating largely stimulates the compulsive buying of unhealthy foodstuffs [17], leading to an increased occurrence of specific neurodegenerative and inflammatory psychiatric and physical health comorbidities, such as MDD [18]. MDD shares four gene modules causally associated with PTSD: UBA7, HLA-F, HSPA1B, and RERE [24]. Meanwhile, people with comorbid PTSD-MDD express increased symptom severity and a greater occurrence of hypertension and a high BMI [12,13]. Simultaneously, PTSD with comorbid obesity is associated with the increased likelihood of mental and physical health problems, such as MDD, suicidality, nicotine dependence, food addiction, diabetes, hypertension, insomnia, migraine, and cognitive deficits (poor attention and processing speed) [1,25,26]. Cardiovascular and metabolic dysfunctions in PTSD patients may have devastating effects, including premature mortality, and irreversible mental conditions, such as dementia. Because pharmacological and cognitive behavioral treatments of PTSD may mitigate the risk for cardiometabolic diseases, careful identification of cardiometabolic alterations and their risk factors in PTSD patients may be necessary for tailoring effective treatment programs [27].

Being in close contact with infected COVID-19 patients, frontline healthcare workers (HCWs) have been reported to exhibit a higher prevalence of PTSD than infected patients [28]. This is possibly due to their perceived susceptibility to contracting infection along with vicarious trauma, which may stem from witnessing patients die unexpectedly or endure severe suffering [29]. Those who are females, less experienced, or working in closed units with difficult-to-manage conditions express the highest levels of trauma and burnout, especially when they lack social support [30]. The risks of COVID-19 and related fear of infection are high in dentistry because of its special nature—operating in the mouth entails a high risk of exposure to droplets from asymptomatic patients [31,32]. HCWs hospitalized because of contracting COVID-19 infection are those with an advanced age and comorbidities (e.g., diabetes, obesity, and hypertension) [33]. Therefore, HCWs with such comorbidities may have higher perceived susceptibility to COVID-19 and consequently be more prone to PTSD, rendering them worthy of an evaluation for PTSD symptomatology.

The Impact of Event Scale-Revised (IES-R) is a famous measure of PTSD, which corresponds to PTSD’s main criteria (intrusion, avoidance, and arousal) that were plotted in the earlier versions of the DSM. In different studies, the IES-R demonstrated various structures, ranging from a single dimension to six dimensions (reviewed in [34]). PTSD criteria have been further expanded in the most recent version of the DSM according to the latest research findings [2,3,35], which may support the credibility of the multi-dimensional structures of the IES-R. The six-dimension structure of the IES-R has been reported recently in two Arab samples of healthy adults and psychiatric patients, and it expressed superior construct validity relevant to previously reported structures. Its subscales demonstrated good internal consistency, discriminant validity, convergent validity, and criterion validity, which denotes their usefulness for revealing people with higher levels of mental distress [34,36]. The concurrent validity of this structure has not been tested yet, with a possibility that its psychometric qualities may considerably vary in a different cultural context since Arabs may be limited in interpreting/appraising trauma and expressing negative emotions than people from western cultures [37]. This study expands the existing knowledge on the characteristics of the six-dimensional structure of the IES-R in a sample of Russian dental HCWs during the COVID-19 pandemic. As per the existing literature, it examined the probability of having IES-R structures with more than three dimensions in the present sample. It tested the internal consistency as well as the convergent, concurrent, and criterion validity of the six-dimension structure of the IES-R relative to the theory-based three-dimension structure. We hypothesized that: (1) the six-dimension structure of the IES-R expresses sound concurrent validity by strongly correlating with another measure of PTSD that is same as the three-dimension structure, and (2) both IES-R structures demonstrate sufficient criterion validity, as they may be causally linked to elevated BMI and hypertension.

## 2. Materials and Methods

### 2.1. Study Design and Participants

This instrumental study employed a cross-sectional design and recruited a convenience sample of dental HCWs (N = 58) from two emergency hospitals in Ekaterinburg in the Russian Federation during the period between the 1st and 20th of September 2020. The study included dental frontline HCWs who consented to participate in the study and who were working before January 2020 and continued to work after that. HCWs were excluded if they refused to take part or were on sick leave or maternity/parental leave [38,39].

We conducted the analysis using data from a public dataset shared under the terms of Creative Common License CC By 4.0 [38]. Ethical approval for the procedure of data collection had been previously granted during meeting # 6 of the Ethics Commission of the Academic Council of the Chelyabinsk State University (Russia) [39]. Because an ethical agreement was already in place, we assessed the data in the current analysis.

### 2.2. Measures

Participants self-reported data via a test battery that gathered information on their sociodemographic characteristics (e.g., age, gender, and work category), weight, hypertension diagnosis, smoking habits, and potential COVID-19 symptoms (e.g., high fever, fatigue, and muscle aches). The test battery also included two measures of PTSD.

1. Posttraumatic Stress Disorder Symptom Scale-Self Report (PSS-SR) is a valid tool for evaluating PTSD diagnosis and its severity. It comprises 17 items in 3 subscales, which identify three key PTSD symptoms: re-experiencing (items 1 to 5), avoidance (items 6 to 12), and hyperarousal (items 13 to 17). The respondents rated the items of the PSS-SR on 4-point equal response intervals ranging from 0 (not at all) to 3 (almost always). A cutoff score of 23 on the PSS-SR was identified as optimal for indicating the potential for a PTSD diagnosis [40]. The reliability of the PSS-SR and its three subscales in the current sample is excellent or good (alpha = 0.92, 0.88, 0.86, and 0.76, respectively).
2. The Impact of Event Scale-Revised (IES-R) is a 22-item measure of the level of subjective distress associated with specific traumatic exposure, herein direct exposure to possible COVID-19 infection. It consists of three subscales, herein referred to as IES-R3, which are labeled as intrusion (e.g., intrusive thoughts/feelings/imagery and nightmares: items 1, 2, 3, 6, 9, 14, 16, and 20), avoidance (e.g., avoidance of feelings/situations/ideas/memories: items 5, 7, 8, 11, 12, 13, 17, and 22), and hyperarousal (e.g., anger/irritability and hypervigilance/heightened startle/poor concentration: items 4, 10, 15, 18, 19, and 21) [39]. The six-dimension structure of the IES-R, herein referred to as IES-R6, are avoidance (items 5, 8, 11, 17, and 22), intrusion (items 1, 3, 6, 9, and 20), numbing (e.g., numbing of responsiveness: items 7, 12, 13, and 14), hyperarousal (e.g., physical reactions and being on guard: items 16, 18, 19, and 21), sleep problems/disturbance (e.g., trouble falling and staying asleep: items 2 and 15), and irritability/dysphoria (e.g., anger and irritability: items 4 and 10) [36]. The respondents rated the items of the IES-R on 5-point equal response intervals ranging from 0 (not at all) to 4 (extremely) [39].

### 2.3. Statistical Analysis

Categorical variables were reported as frequency and percentage. Quantitative variables with normal and non-normal distributions were reported as mean ± standard deviation and median (interquartile range: Q1–Q3), respectively. A preliminary investigation of the structure of the IES-R was conducted through an exploratory factor analysis (EFA: maximum likelihood, direct Oblimin rotation, Kaiser–Meyer–Olkin (KMO) measure of sampling adequacy, and Bartlett’s test of sphericity). The reliability of the IES-R and its subscales was assessed using coefficient alpha. Convergent validity was evaluated through item total correlations and the correlations between the three- and six-subscale structures and the total IES-R score. Concurrent validity was examined by correlating the IES-R and its subscales with the PSS-SR and its subscales. Criterion validity was tested by correlating the IES-R and its subscales with BMI and hypertension diagnoses.

Receiver operating characteristic (ROC) curve represents a reliable computational method, which illustrates the ability of a risk prediction model to distinguish between individuals with and without a condition by determining clinically relevant thresholds. The curve plots the true-positive rate (TPR or sensitivity) against the false-positive rate (FPR or specificity) across all thresholds (typically at selected intervals). Each point on the ROC curve corresponds to a specific sensitivity and specificity pair [32,41]. We ran an ROC curve analysis using the two IES-R structures to predict the PSS-SR, hypertension diagnosis, and high BMI. For this test, the IES-R and its subscales were used as continuous variables. In the meantime, the participants were categorized according to the reported cutoff of the PSS-SR into two categories (23 or below: no PTSD, and above 23: possible PTSD) [40] as well as BMI (below 25: normal weight, and 25 or above: overweight/obesity). This test was intended to examine the cutoff scores of the IES-R and its three/six subscales, which might distinguish those with PTSD, high BMI, and hypertension. The diagnostic accuracy of the ROC model corresponds to the values of the area under curve (AUC), sensitivity and specificity for all possible cut points, and the Youden index—the sum of sensitivity and specificity of the optimal point minus one. In ideal tests, all the values should be close to one [41]. 

Two-step cluster analysis is one of the most reliable classification techniques in terms of the classification probability of individuals/data items into subgroups, the number of subclusters identified, and reproducibility of the results on clinical and other types of data. It is a hybrid approach, which first uses a distance measure to separate the raw input data into a manageable set of subclusters. In the second step, it uses a hierarchical clustering method to progressively merge homogenous subclusters into larger clusters, ending with the selection of the optimal subgroup model [41,42]. Two-step cluster analysis was conducted using the IES-R, PSS-SR, and their dimensions to classify the participants according to BMI and hypertension. Model fit was determined based on the silhouette measure of cohesion and separation; ideally it should be greater than 0.5 and closer to 1. Meanwhile, the best cluster classification solution was determined based on Akaike Information Criterion (AIC); the cluster solution with the lowest AIC is the best solution [42].

Two path analysis models were conducted using the three and six dimensions of the IES-R as independent variables to predict the variance in BMI and hypertension. The analysis was adjusted for the confounding effects of age, gender, and current smoking. The goodness of model fit was considered based on chi square χ^2^/DF (CMIN/DF) less than 3, as well as the Root Mean Square Error of Approximation (RMSEA) and Standardized Root Mean Square Residual (SRMR) less than 0.06, along with the comparative fit index (CFI), and Tucker–Lewis index (TLI) greater than 0.95 [43]. Most non-significant paths were trimmed to improve the model fit. The analyses were performed with SPSS version 28 and Amos version 26, with significance considered at a level below 0.05 in two-tailed tests.

## 3. Results

Females were a majority (n = 49, 82.8%), with an average age of 44.1 ± 12.2, range = 22–71 years. HCWs in this sample had a median work experience (interquartile range: Q1–Q3) of 16.0 (5.0–30.0) years, and they were categorized according to their position into three classes: (1) dental auxiliaries, such as dental laboratory technicians, front desk receptionists, and nurse aides (n = 31, 53.5%); (2) dentists with an MD degree (n = 17, 29.3%); and (3) dental assistants, HCWs without an MD degree (n = 10, 17.2%). According to the cutoff of the PSS-SR, only two participants were classified as probable cases of PTSD. 

EFA analysis revealed that the structure of the IES-R covers five factors with eigenvalues greater than one, which explain 76.0% of the variance (Supplementary Materials). Despite the small size of the present sample, the values of the KMO (0.79) and Bartlett’s (*χ*^2^(231) = 1154.61, *p* < 0.001) tests were sufficiently high, suggesting the suitability of the sample for EFA. The reliability of the IES-R was excellent (alpha = 0.95), with item total correlations ranging from 0.48 to 0.83 and alpha if the item was deleted ranging from 0.946 to 0.951. The reliability of the subscales of the IES-R3 and the IES-R6 was very good for the former and ranged from acceptable to very good for the latter (Table 1). The values of item total correlations were high, especially for the six subscales, which indicates adequate convergent validity.

A detailed description of the IES-R, PSS-SR, their scales, BMI, and the frequency of hypertension and smoking is shown in Table 2, which also shows that the three and six subscales of the IES-R strongly correlate with the parent scale as well as with each other, denoting good convergent validity. They also strongly correlate with the PSS-SR and its three subscales, suggesting good concurrent validity, as hypothesized. Only the correlation between the irritability dimension and the reexperiencing subscale of the PSS-SR is not significant.

As for tests of criterion validity, neither the PSS-SR nor its subscales correlated with BMI or hypertension. The correlations of the IES-R with BMI and hypertension were also non-significant, contrary to the expectations (Table 2). However, BMI expressed significant positive correlations with the intrusion and hyperarousal dimensions of IES-R3 and IES-R6. Having a diagnosis of hypertension significantly correlated with the avoidance and intrusion dimensions of IES-R3 as well as the avoidance, numbing, and hyperarousal dimensions of IES-R6. The PSS-SR and its avoidance subscale correlated with current smoking, while only the irritability subscale of the IES-R correlated with this variable.

**Figure 1:**
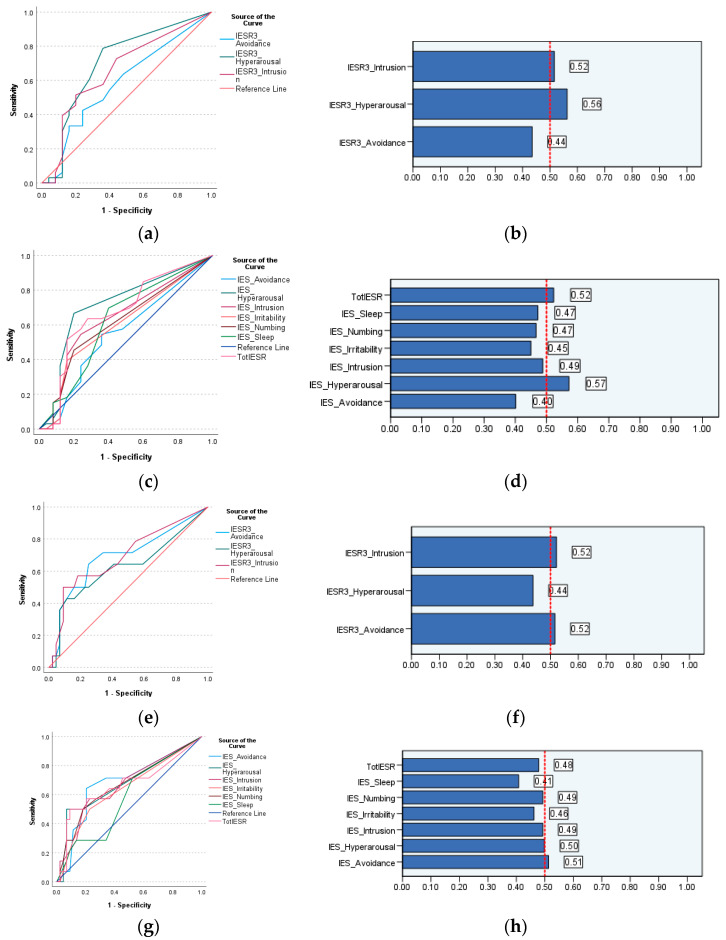
Receiver operating characteristic (ROC) curve and quality of models using the scores of the three and six dimensions of the Impact of Event Scale-Revised (IES-R) to classify dental healthcare workers according to their BMI (**a**–**d**) and history of hypertension (**e**–**h**).

ROC analysis demonstrated excellent diagnostic accuracy for the IES-R (with a cutoff of ≥39.5) and its three- and six-dimensional models (at different cutoff points, see Table 3) in identifying PTSD as indicated by the PSS-SR. All AUC values and AUC 95% confidence intervals were above 0.9. Both models showed an excellent fit, as illustrated in Supplementary Figures S2 and S4, with significance at the 0.001 level. ROC analysis also revealed significant associations of the IES-R and IES-R3 intrusion dimension (*p* = 0.023, 0.030) with high BMI, while both hyperarousal subscales expressed good diagnostic accuracy for overweight/obesity (both AUC values = 0.71), with slight variations in specificity and sensitivity between subscales from the three- and six-dimension IES-R structures. While the IES-R was not associated with hypertension (*p* = 0.083), the avoidance and intrusion subscales of IES-R3 as well as the avoidance subscale of IES-R6 were significantly (*p* = 0.033, 0.027, 0.035, respectively) associated with hypertension, and its association with IES-R6 hyperarousal was marginally significant (*p* = 0.052). The values of AUC and the Youden index (Table 3) indicate the fair diagnostic accuracy of most significant ROC models for BMI and hypertension. Figure 1 indicates that the significant models demonstrate a marginally acceptable quality.

**Figure 2:**
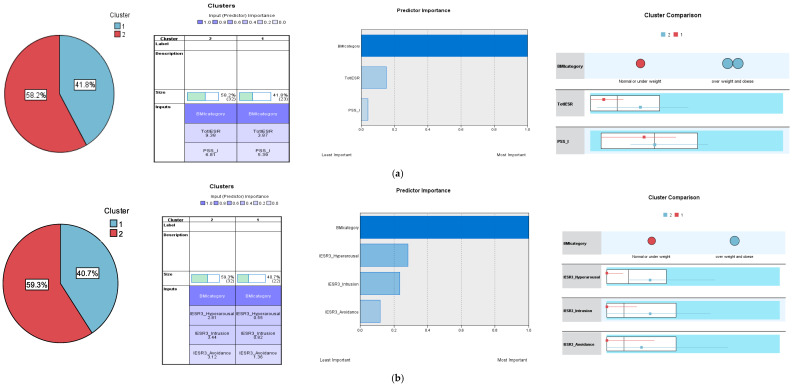

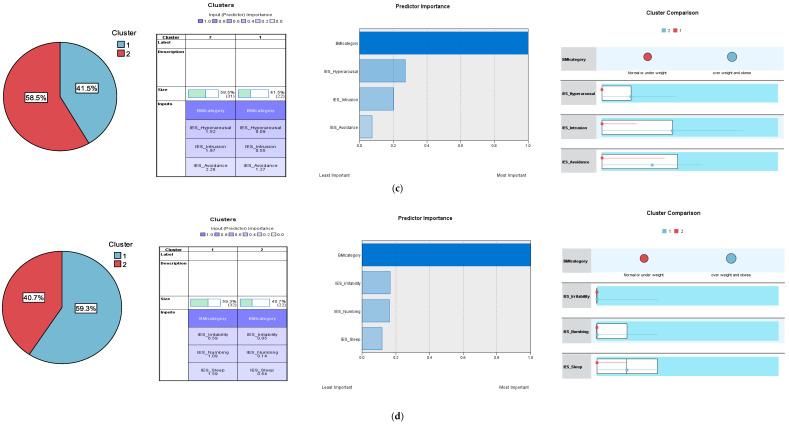

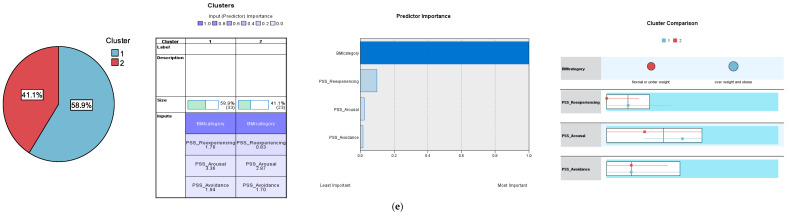
Cluster analysis using the Impact of Event Scale-Revised (IES-R); Posttraumatic Stress Disorder Symptom Scale-Self-Report (PSS-SR), and their subscales to classify the participants according to their body mass index (BMI). (**a**) Impact of Event Scale-Revised (IES-R) and Posttraumatic Stress Disorder Symptom Scale-Self-Report (PSS-SR). (**b**) The three-dimension structure of the Impact of Event Scale-Revised (IES-R3): hyperarousal, intrusion, and avoidance. (**c**) Hyperarousal, intrusion, and avoidance dimensions of the six-dimension structure of the Impact of Event Scale-Revised (IES-R6). (**d**) Numbness, sleep, and irritability dimensions of the six-dimension structure of the Impact of Event Scale-Revised (IES-R6). (**e**) Avoidance, arousal, and re-experiencing dimensions of the Posttraumatic Stress Disorder Symptom Scale-Self Report (PSS-SR).

Two-step cluster analyses with five sets of trauma input variables [(IES-R, PSS-SR), (intrusion, hyperarousal, and avoidance dimensions of IES-R3 and IES-R6), (numbing, sleep disturbance, and irritability dimensions of IES-R6), and (avoidance, arousal, and re-experiencing dimensions of the PSS-SR)] converged on revealing the presence of two separate clusters (normal weight/low BMI and overweight/obese) as the most efficient and robust classification of the participants according to their body composition. The values of the silhouette measure of cohesion and separation (around 0.5) indicate that the IES-R, PSS-SR, and their subscales can fairly classify the sample, while AIC values (Supplementary Materials S2: Supplementary Table S4) confirmed the two-cluster classification as the best solution. Model comparisons (Figure 2) show persistently higher levels of all trauma variables (except for the numbing and irritability dimensions of IES-R6) among participants in the overweight/obese cluster than in the normal weight cluster. As shown in Figure 2 (predictor importance charts) and Supplementary Materials S2: (Supplementary Table S5), the highest predictor importance was recorded for the hyperarousal and intrusion subscales of both IES-R structures followed by the irritability and numbing subscales of IES-R6, then the IES-R (range = 15–28%). On the contrary, the PSS-SR and its three subscales witnessed the lowest predictor importance (range = 1–9%).

**Figure 3:**
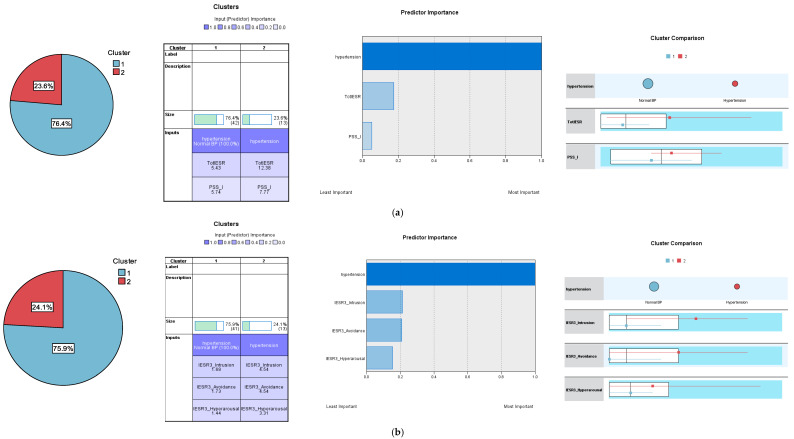
Cluster analysis using the Impact of Event Scale-Revised (IES-R), Posttraumatic Stress Disorder Symptom Scale-Self Report (PSS-SR), and their subscales to classify the participants according to the diagnosis of hypertension. (**a**) Impact of Event Scale-Revised (IES-R) and Posttraumatic Stress Disorder Symptom Scale-Self Report (PSS-SR). (**b**) The three-dimension structure of the Impact of Event Scale-Revised (IES-R3): hyperarousal, intrusion, and avoidance.

Using the same five sets of trauma input variables to classify the participants according to the diagnosis of hypertension converged on revealing the presence of two separate clusters (normal blood pressure and hypertension) as the most efficient and robust classification only in two tests, which comprised the IES-R and the PSS-SR as well as the intrusion, hyperarousal, and avoidance dimensions of IES-R3 (Figure 3). One cluster solution emerged in the rest of the tests. Silhouette measure indicated humble model fit, and the AIC with the lowest values appeared only in the two-cluster models (Supplementary Materials S2: Supplementary Table S6) while the highest predictor importance was recorded for the intrusion and avoidance subscales followed by the IES-R (Supplementary Materials S2: Supplementary Table S7).

**Figure 4:**
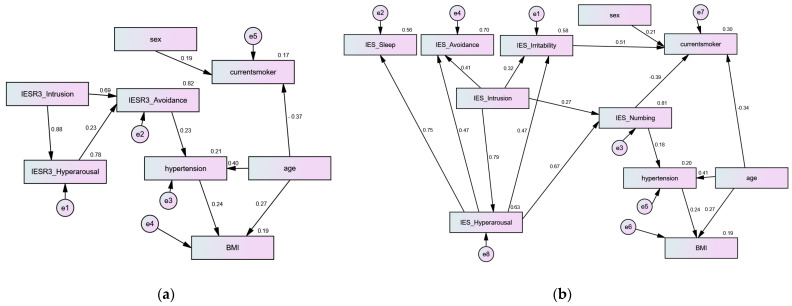
Path analysis model using the three and six dimensions of the Impact of Event Scale-Revised (IES-R: (**a**) and (**b**), respectively) to predict hypertension, body mass index (BMI), and current smoking status.

The fit of both path analysis models was excellent (χ^2^ = 10.17, 24.57; DF = 19, 39; *p* = 0.949, 0.965; CMIN/DF = 0.54, 0.63; both CFI values = 0.999, both TLI values = 0.999, both RMSEA values = 0.000; SRMR = 0.074, 0.078)—the SRMR value fell within the acceptable, less conservative threshold of <0.08. The model comprising IES-R3 (Figure 4a) predicted 21, 19, and 17% of the variances in hypertension, BMI, and current smoking, respectively, while the model comprising IES-R6 (Figure 4b) predicted 20, 19, and 30% of the variances in hypertension, BMI, and current smoking, respectively. In both models, age was a significant predictor of the three key outcomes (*p* = 0.001, 0.036, and 0.002), while hypertension was a significant predictor of BMI, but not the opposite. Gender was not significantly associated with any of the outcome variables in either model, albeit it had a marginally significant effect on current smoking in model b only (*p* = 0.058). In both models, none of the PTSD symptoms of either IES-R structure significantly contributed to the variance in hypertension and BMI either directly or indirectly. Likewise, none of the IES-R3 dimensions contributed to current smoking, while the irritability and numbing subscales of IES-R6 were significantly associated with current smoking. However, current smoking was not significantly associated with BMI and hypertension in either model. Irritability scores were significantly higher among smokers (Mann–Whitney U = 158.0, z = −2.10, *p* = 0.035), while the difference in numbing between smokers and non-smokers was non-significant (*p* = 0.129).

## 4. Discussion

Despite being introduced over three decades ago, DSM criteria for PTSD continue to be refined as the research evolves. Similar to reports of more than three factors for the IES-R in war and fire survivors [44,45], the six-dimensional IES-R model was recently proposed as a rigorous model in two Arab samples [34,36]. However, this structure has yet to be tested in other populations or cultures. This study supports the plausibility of this structure among Russian healthcare workers in the context of the COVID-19 pandemic as EFA, which applies no constraints, revealed a five-factor IES-R structure. Despite the considerably small sample size, both IES-R structures expressed high internal consistency and convergent validity as noted by high values of item total correlations, as well as strong correlations of the subscales with the parent scale and the corresponding original subscales of the three-dimension IES-R3 (Table 1)—both criteria reflect high cohesion among the items of the scale and its subscales [46]. The IES-R3 and the IES-R6 demonstrated excellent concurrent validity as noted by strong positive correlations with the PSS-SR and its subscales (all *p* values < 0.01)—the only non-significant correlation was that of the irritability subscale of IES-R6 and the re-experiencing subscale of the PSS-SR. In the ROC analysis, the IES-R and its two structures significantly predicted PTSD symptoms measured by the PSS-SR, with all AUC values above 0.9. These correlational and predictive patterns support the concurrent validity of both IES-R structures. Nonetheless, the tests of criterion validity resulted in mixed findings, which are discussed in detail below.

We refrained from investigating the structure of IES-R6 through confirmatory factor analysis and multigroup analysis due to the non-normal distribution of the IES-R and the small sample size relative to the number of its items. Nevertheless, using trauma variables of the IES-R, the PSS-SR, and their subscales as independent/input variables in ROC and two-step cluster analyses divided the total sample into two groups or clusters, which were generally determined as normal weight vs overweight/obese and normal blood pressure vs hypertensive. While the IES-R was strongly associated with the PSS-SR and its subscales, correlations and classification models suggest the stronger association of the IES-R and its subscales with BMI and hypertension than all associations expressed by the PSS-SR and its subscales—(1) ROC models using the PSS-SR and its three subscales to predict BMI and hypertension were all non-significant and expressed a poor fit, with AUC values ranging from 0.53 to 0.66 (Supplementary Materials S1: Tables S2 and S3, Supplementary Figures S5–S8), and (2) predictor importance in two-step cluster models using the PSS-SR and its subscales ranged from 1 to 9%, while the importance of the IES-R and its subscales from both structures ranged between 12 and 28% (Supplementary Materials S2). Accordingly, the IES-R and its two structures may be more favorable than the PSS-SR as criterion variables in studies examining the link between trauma exposure and cardiometabolic alterations.

Consistent with previous studies [16,20,47], intrusion and hyperarousal correlated with high BMI in our participants (Table 1) and classified them according to their BMI into two distinct clusters in which overweight/obese participants displayed greater levels of intrusion and hyperarousal (Figure 2: cluster comparison charts). Herein, we attempted to offer a mechanism through which certain IES-R components may relate to cardiometabolic comorbidities in PTSD. Obesity in individuals with PTSD is linked to disordered eating behaviors, such as binge eating [8]. Experiential avoidance of trauma-related memories/stimuli may play a role in this relation [48], which may operate through rumination—similar to that expressed in the stress reaction in PTSD-related CVD risk. In this respect, overweight and obese people exhibit an impaired ability to inhibit intrusive thoughts about food and automatic or dominant eating behaviors [49,50,51], particularly because of their higher levels of poor impulse control (urgency, lack of perseverance, and sensitivity to reward) [49]. Indeed, obesity is also associated with food addiction, which takes the form of an obsession with and uncontrolled intake of unhealthy diets (e.g., sugar-rich and ultra-processed foods) despite the negative effects of these foods on physical health. The role of intrusive thoughts is pivotal as obesity increases people’s tendency to a range of addictive behaviors, including binge eating disorders and the intake of illicit drugs [51]. Thus, avoidance in PTSD may activate rumination and subsequent hyperarousal, resulting in a chronic stress-related behavioral and neurophysiological adaptation through which disordered food intake and increased BMI are promoted [52]. The bidirectional roles of cytokines and adipokines in both obesity and PTSD should be also acknowledged [12,13,24].

In our study, hypertension significantly correlated with avoidance and intrusion (IES-R3) as well as with avoidance, numbing, and hyperarousal (IES-R6). However, in ROC and two-step cluster analyses, avoidance and intrusion were more strongly associated with hypertension, while hyperarousal had a marginal association—it expressed the lowest predictor importance among the three input variables in cluster analysis (Supplementary Materials S2: Table S7). This result can be interpreted in the light of the available literature. Consistent with our results, in an examination of the effect of different PTSD clusters in 1111 military personnel from the UK, avoidance significantly correlated with systolic blood pressure, intrusion correlated with visceral adiposity, and emotional numbing correlated with the greater estimated glucose disposal rate while hyperarousal correlated with greater levels of triglycerides. In that study, PTSD clusters did not correlate with the inflammatory marker c-reactive protein, indicating the robustness of the cardiometabolic aspect of PTSD over and above the inflammatory [47]. Likewise, PTSD symptomatology and exposure to trauma are associated with greater expressive suppression and less cognitive reappraisal (*p* = 0.02), as emotional regulation strategies, in hypertensive than in normotensive war survivors [12]. Unlike cognitive reappraisal, expressive suppression in individuals exposed to trauma is significantly associated with higher stress-related reactions and related key psychopathologies (PTSD, anxiety, and depression). Rumination partially mediated these associations [4]. The examination of neural activity through functional magnetic resonance imaging among combat-related PTSD patients and combat-exposed controls who were asked to reappraise or suppress their emotional response prior to viewing combat-related images revealed reduced medial prefrontal neural activity during reappraisal and increased prefrontal neural activity during image viewing, with increased arousal ratings in all conditions [5]. Likewise, PTSD re-experiencing is associated with low cerebrospinal fluid levels of the neuroactive steroids, allopregnanolone, and its equipotent enantiomer, pregnanolone (collectively termed ALLO)—3-α-reduced biosynthetic derivatives of progesterone. Such alterations are conducive to fear conditioning in PTSD individuals as well as the HPA axis and sympathetic system reactivity, potentiating the release of cortisol and NPY into visceral fat tissues during severe stress [52]. Therefore, intrusive traumatic thoughts, which are not assimilated due to the defective use of avoidance/expressive suppression, may lead to heightened arousal—an indicator of HPA axis dysregulation, which in turn increases the risk of cardiometabolic diseases.

The new dimensions of the IES-R (numbing, sleep disturbance, and irritability) were largely predicted by the PTSD symptoms of intrusion and hyperarousal (Figure 4b). While these new dimensions had positive associations with BMI and CVDs (Table 1), these associations were less strong than associations possessed by the major three PTSD symptoms (Table 2, Figure 2 and Figure 3), and none of the symptoms was significantly associated with BMI/hypertension in the path analysis. However, only numbing and irritability significantly predicted current smoking (Figure 4b). In line with this, the emotional numbing PTSD cluster was the only significant predictor of lifetime smoking over other PTSD clusters, demographics, and Axis-I comorbidity in the American population. In the same study, the hyperarousal cluster (irritability, hypervigilance, and insomnia) uniquely correlated with nicotine dependence over other PTSD clusters, demographics, and Axis-I comorbidity [35]. In the same way, irritability recorded the highest predictivity among the six dimensions of the IES-R for psychological distress in psychiatric patients and healthy adults [36]. Taken together, the findings call attention to the screening for obesity and cardiovascular risk, as well as high-risk factors for these two conditions (e.g., unhealthy diet and physical inactivity) in people at risk of PTSD. PTSD patients may be further assessed for specific food preferences and impulsivity—an uncontrolled urge to buy unhealthy foods. Accordingly, they may receive personalized targeted nutritional treatment (e.g., abstinence from specific foods or inclusion of persistently avoided foods, such as vegetables) [1,17,53]. Understanding the dynamics through which specific PTSD symptoms may affect food choice and buying preferences may aid in the development of more effective interventional strategies for improving the mental and physical outcomes of PTSD. In this respect, primary care and similar non-stigmatizing community healthcare settings may aid in inducing meaningful and lasting behavior changes among PTSD patients with comorbid cardiometabolic conditions by implementing rigorous clinical practice guidelines, which embed evidenced-based, patient-centered, multicomponent, and combined lifestyle interventions (e.g., counselling, literacy interventions, and self-management that focus on diet, exercise, and complementary therapies) in prevention programs. A solid promotion of behavioral change techniques is necessary to overcome potential challenges through a well-coordinated implementation strategy that involves supporting clinicians in implementing guidelines in everyday practice, offering them without costs, and monitoring their long-term effectiveness [54,55].

The findings of this study may have important clinical implications since the IES-R is a publicly available and free measure, and the scale and all its subscales properly detected PTSD cases identified by another widely used PTSD measure (PSS-SR): the gold standard. Its numerous symptom clusters/subscales seem to possess greater discriminant validity (i.e., for cardiometabolic morbidities) than that of the PSS-SR and its subscales. They may operate interrelatedly to flag obesity, hypertension, and related risk factors, such as smoking. However, the results of our study are just preliminary and should be interpreted with caution. Notably, model quality charts (Figure 1) and the silhouette measure of cohesion and separation (Supplementary Materials S2) indicate poor fit of all the classification tests. Meanwhile, significant associations of the IES-R dimensions with BMI and hypertension disappeared in the path analysis, making room for age as a more influential factor in obesity and hypertension than the dimensions of IES-R3 and IES-R6. Smoking is a documented risk factor for CVDs, and it is associated with BMI through a pathway that involves reduced food intake. However, current smoking failed to predict hypertension/BMI in our path model. 

Our exceptionally small sample size, which was not determined using the power analysis, may reduce the statistical power of our tests and increases the margin of error. This main threat to the internal and external validity of our study marks the findings as preliminary and necessitates the need for replicating the analysis in larger and more diverse samples. Selection bias is another key limitation as females were a majority, and the sample was convenient and homogenous from a single country. Moreover, the recruitment process (the number of HCWs initially targeted, declined to participate, or were excluded) is not clear because we did not collect the data ourselves—we used public data and referred to the available description in the published report, which did not describe the recruitment procedure. Reporting/recall bias is inherent in self-reported data, like in the present study. PTSD as a diagnosis was based on self-reported measures only, with a lack of confirmation of Criterion A in the DSM (i.e., probable exposure to COVID-19 during dental care provision was not confirmed by the respondents as a direct threat) [36], which casts doubt on the credibility of the results, especially as only two PTSD cases exist in the present sample based on the cutoff of the PSS-SR. The absence of some key measures (e.g., mood symptoms) limited our ability to fully explore the interaction of important factors, which may influence the association of PTSD components with cardiometabolic problems. The cross-sectional design is another limitation. Longitudinal data provide more reliable evidence on the direction of the relationship between PTSD and obesity/hypertension [16]. The literature documents the contribution of PTSD to unhealthy behaviors conducive to both conditions [21]. However, in our study, only smoking was assessed, and it was not associated with any trauma variable except the irritability and numbing subscales. Therefore, future studies may consider whether the IES-R may reflect more directly related risk factors such as the intake of an improper diet and lack of physical activity. Our study only reported overall obesity (BMI), while central/abdominal obesity may exist in people with a normal BMI. A meta-analysis reports a prevalence of abdominal obesity in 49.3% (95% CI = 29.7–69.0%) of PTSD victims [56]. Meanwhile, longitudinal data show significant associations of abdominal obesity with an increased risk of CVDs and all-cause mortality, even among young people with normal weight [57]. In addition to weight control, guideline designers should provide recommendations for people to decrease abdominal fat accumulation in their effort to reduce mortality risk in later life [57]. Therefore, future studies may address the diagnostic accuracy of PTSD measures for both general and central obesity. Further investigations of IES-R6 in larger samples of different conditions from various countries are still needed.

## 5. Conclusions

The findings demonstrate the high internal consistency and strong convergent and concurrent validity of the freely available IES-R. Both the established three-dimension structure and the newer six-dimension structure showed significant correlations with and excellent predictive ability for PTSD symptoms measured by the PSS-SR. The IES-R and some of its subscales (e.g., intrusion, avoidance, and hyperarousal) may serve as useful criterion variables for identifying PTSD-related cardiometabolic effects, as they exhibited a better diagnostic capacity for high BMI and hypertension than the PSS-SR and its subscales. However, the diagnostic accuracy of these measures was modest, and the results should be interpreted cautiously, given the small sample size. Emotional subscales of the IES-R, such as irritability and numbing, were linked to a higher tendency to smoke—a potential behavioral correlate of cardiovascular and metabolic dysfunction. Therefore, investigating the role of specific PTSD symptoms in influencing food preferences and lifestyle behaviors associated with obesity and CVDs could be important for developing targeted treatment strategies. Replicating the study in larger, more diverse samples from various settings and cultural contexts is recommended.

## Figures and Tables

**Table 1:** Internal consistency of the Impact of Event Scale-Revised (IES-R) and its three and six subscales.

	Alpha	Alpha if Item Deleted	Item Total Correlations
IES-R	0.95	0.946 to 0.951	0.48 to 0.83
T-Avoidance	0.84	0.81 to 0.84	0.46 to 0.64
T-Intrusion	0.88	0.85 to 0.88	0.46 to 0.85
T-Hyperarousal	0.86	0.82 to 0.88	0.39 to 0.81
S-Avoidance	0.75	0.67 to 0.72	0.46 to 0.59
S-Intrusion	0.81	0.72 to 0.80	0.47 to 0.77
S-Numbing	0.83	0.72 to 0.87	0.48 to 0.80
S-Hyperarousal	0.88	0.67 to 0.72	0.81 to 0.87
S-Sleep	0.84	-	0.72
S-Irritability	0.69	-	0.52

T-: Three-dimension structure of the of the IES-R; S-: six-dimension structure of the of the IES-R.

**Table 2:** Convergent validity, concurrent validity, criterion validity, and descriptive statistics of the three and six subscales of the Impact of Event Scale-Revised (IES-R).

Variables	1	2	3	4	5	6	7	8	9	10	11	12	13	14	15	16	17
1. IES-R	--																
2. T-Avoidance	0.916 **	--															
3. T-Intrusion	0.903 **	0.792 **	--														
4. T-Hyperarousal	0.893 **	0.721 **	0.768 **	--													
5. S-Avoidance	0.870 **	0.977 **	0.727 **	0.668 **	--												
6. S-Intrusion	0.861 **	0.786 **	0.960 **	0.706 **	0.733 **	--											
7. S-Numbing	0.788 **	0.783 **	0.788 **	0.710 **	0.680 **	0.723 **	--										
8. S-Hyperarousal	0.878 **	0.804 **	0.793 **	0.892 **	0.764 **	0.753 **	0.761 **	--									
9. S-Sleep	0.797 **	0.604 **	0.704 **	0.870 **	0.560 **	0.591 **	0.631 **	0.693 **	--								
10. S-Irritability	0.621 **	0.528 **	0.595 **	0.684 **	0.485 **	0.558 **	0.526 **	0.627 **	0.449 **	--							
11. PSS-SR	0.642 **	0.516 **	0.692 **	0.573 **	0.486 **	0.619 **	0.542 **	0.579 **	0.589 **	0.416 **	--						
12. PSS_Avoidance	0.545 **	0.463 **	0.586 **	0.414 **	0.453 **	0.576 **	0.402 **	0.419 **	0.416 **	0.375 **	0.879 **	--					
13. PSS_Arousal	0.630 **	0.469 **	0.646 **	0.644 **	0.432 **	0.570 **	0.510 **	0.568 **	0.658 **	0.471 **	0.904 **	0.719 **	--				
14. PSS_Reexperiencing	0.494 **	0.437 **	0.576 **	0.393 **	0.404 **	0.501 **	0.514 **	0.479 **	0.411 **	0.190	0.773 **	0.587 **	0.564 **	--			
15. BMI	0.250	0.185	0.303 *	0.276 *	0.146	0.299 *	0.240	0.390 **	0.106	0.205	0.009	−0.008	−0.041	0.200	--		
16. Hypertension	0.245	0.295 *	0.294 *	0.193	0.286 *	0.257	0.293 *	0.287 *	0.123	0.253	0.177	0.166	0.076	0.251	0.344 **	--	
17. Smoking	0.034	−0.074	0.038	0.040	−0.049	0.019	−0.201	0.037	−0.056	0.279 *	0.279 *	0.328 *	0.242	0.125	−0.116	−0.151	--
Median	5.0	1.0	1.0	1.0	1.0	1.0	0	0	1.0	0	6.0	1.0	1.0	1.0	26.8▪	14•	10•
Interquartile range: Q1–Q3	0–13.0	0–6.0	0–5.3	0–4.0	0–5.0	0–4.0	0–2.0	0–2.0	0–2.0	0–1.0	1–11.0	0–6.3	0–5.3	0–4.0	5.2▪	24.1%•	17.2%•

T-: Three-dimensional structure of the IES-R, S-: six-dimensional structure of the IES-R, PSS-SR: Posttraumatic Stress Disorder Symptom Scale-Self Report, BMI: body mass index, ▪: results are reported as mean ± SD, and •: results are reported as number and percentage; * *p* < 0.05; ** *p* < 0.01

**Table 3:** Cutoff scores of the Impact of Event Scale-Revised (IES-R) and its subscales, along with goodness-of-fit indices associated with receiver operating characteristic (ROC) curve analysis in dental healthcare workers.

	Outcome Variables	AUC	*SE*	AUC 95% CI	Cutoff	Sensitivity	Specificity	Youden Index
IES-R	PSS-SR	1.00	0.00	0.98 to 1.01	39.5	1.00	1.00	1.00
	Obesity	0.67	0.08	0.52 to 0.82	5.5	0.64	0.72	0.36
	Hypertension	0.66	0.10	0.48 to 0.85	17.5	0.50	0.91	0.41
IES-R3-Avoidance	PSS-SR	1.00	0.00	1.00 to 1.00	12.5	1.00	1.00	1.00
	Obesity	0.59	0.08	0.44 to 0.74	3.5	0.42	0.76	0.18
	Hypertension	0.69	0.09	0.52 to 0.87	3.5	0.57	0.73	0.30
IES-R3-Intrusion	PSS-SR	1.00	0.00	1.00 to 1.00	14.5	1.00	1.00	1.00
	Obesity	0.66	0.07	0.52 to 0.81	2.5	0.52	0.80	0.32
	Hypertension	0.69	0.09	0.52 to 0.86	6.5	0.50	0.91	0.41
IES-R3-Hyperarousal	PSS-SR	0.99	0.01	0.97 to 1.01	10.5	1.00	0.98	0.98
	Obesity	0.71	0.07	0.56 to 0.84	0.5	0.79	0.64	0.43
	Hypertension	0.63	0.10	0.44 to 0.81	6.5	0.43	0.87	0.32
IES-R6-Avoidance	PSS-SR	1.00	0.008	0.98 to 1.01	7.5	1.00	0.98	0.98
	Obesity	0.55	0.08	0.40 to 0.71	1.5	0.55	0.64	0.19
	Hypertension	0.68	0.09	0.51 to 0.85	3.5	0.64	0.80	0.44
IES-R6-Intrusion	PSS-SR	1.00	0.00	1.00 to 1.00	8.5	1.00	1.00	1.00
	Obesity	0.64	0.08	0.49 to 0.79	1.5	0.55	0.76	0.31
	Hypertension	0.66	0.09	0.49 to 0.84	2.5	0.57	0.77	0.34
IES-R6-Numbing	PSS-SR	1.00	0.00	1.00 to 1.00	6.5	1.00	1.00	1.00
	Obesity	0.62	0.08	0.47 to 0.76	0.5	0.46	0.80	0.26
	Hypertension	0.66	0.09	0.49 to 0.84	1.5	0.50	0.81	0.31
IES-R6-Hyperarousal	PSS-SR	0.99	0.02	0.96 to 1.01	7.0	1.00	0.98	0.98
	Obesity	0.71	0.07	0.57 to 0.85	0.5	0.67	0.80	0.47
	Hypertension	0.68	0.09	0.50 to 0.86	3.5	0.29	0.93	0.22
IES-R6-Sleep	PSS-SR	0.98	0.02	0.93 to 1.01	5.5	1.00	0.95	0.95
	Obesity	0.62	0.08	0.47 to 0.77	0.5	0.70	0.60	0.30
	Hypertension	0.58	0.09	0.41 to 0.75	0.5	0.71	0.48	0.19
IES-R6-Irritability	PSS-SR	0.99	0.02	0.95 to 1.02	2.5	1.00	0.95	0.95
	Obesity	0.60	0.08	0.45 to 0.75	0.5	0.39	0.84	0.23
	Hypertension	0.64	0.09	0.46 to 0.81	0.5	0.50	0.77	0.27

PSS-SR: Posttraumatic Stress Disorder Symptom Scale-Self Report.

## Data Availability

The dataset supporting the conclusions of this article is available from the Mendeley repository [38], [https://data.mendeley.com/datasets/gdcmgkcf88] (accessed on 10 September 2024), and also the datasets used and/or analyzed during the current study are available from the corresponding authors on reasonable request.

## Supplementary Materials

The following supporting information can be downloaded at https://www.mdpi.com/article/10.3390/jcm13206045/s1: Supplementary Materials S1: Output of EFA, ROC analyses using the IES-R dimensions to predict the PSS-SR and its subscales, ROC analyses using the PSS-SR and its subscales to predict BMI and hypertension, and detailed output of two-step cluster analyses, including single trauma variables as input variables; and Supplementary Materials S2: two-step cluster model fit and cluster characteristics using five sets of trauma variables to classify the participants according to their BMI and diagnosis of hypertension.
