# Supplementary material for: Cardiometabolic Morbidity (Obesity and Hypertension) in PTSD: A Preliminary Investigation of the Validity of Two Structures of the Impact of Event Scale-Revised
Source: J Clin Med. 2024 Oct 10;13(20):6045. doi: 10.3390/jcm13206045 (PMC11509123; doi:10.3390/jcm13206045)
Supplement: Supplementary file 1 [file jcm-13-06045-s001.zip › jcm-3153571-supplementary/Supplementary Materials S2 Twostep cluster model fit and cluster characteristics.pdf]

Supplementary Materials S2: Twostep cluster model fit and cluster characteristics

Supplementary Table S4: Twostep cluster model fit of trauma variables and body mass index (BMI)

**Input variables**	Obesity			
	**Clusters**	**AIC**	**Silhoutte**	**Ratio of sizes: largest to smallest cluster**
IES-R<br>PSS-SR	1	28.6		
	**2**	**20.6**	0.51	1.39
	3	30.0		
	4	40.0		
IESR3_Intrusion<br>IESR3_Hyperarousal<br>IESR3_Avoidance	1	33.1		
	**2**	**28.0**	0.46	1.45
IESR6_Intrusion<br>IESR6_Hyperarousal<br>IESR6_Avoidance	1	40.9		
	2	28.0	0.42	1.41
IESR6_Numbing<br>IESR6_Irritability<br>IESR6_Sleep	1	42.8		
	**2**	**28.0**	0.52	1.45
PSS_Avoidance<br>PSS_Arousal<br>PSS_Reexperiencing	1	48.9		
	**2**	**46.6**	0.41	1.43
	3	61.0		
	4	80.9		

Supplementary Table S5: Cluster characteristics of Twostep cluster involving trauma variables and body mass index (BMI)

Input variables	Criteria	BMI		Predictor Importance (%)
		**Cluster1** (Normal/underweight)	**Cluster2** (Overweight/obese)	
IES-R<br>PSS-SR	Cluster distribution: Frequency (%)	23 (41.8%)	32 (58.2%)	--
	IES-R	3.9 (6.4)	9.4 (8.5)	15
	PSS-SR	5.4 (5.0)	6.8 (5.2)	3
IESR3_Intrusion<br>IESR3_Hyperarousal<br>IESR3_Avoidance	Cluster distribution: Frequency (%)	22 (37.9)	32 (55.2)	--
	IESR3_Intrusion	0.8 (1.3)	3.4 (3.3)	24
	IESR3_Hyperarousal	0.5 (1.1)	2.8 (2.6)	28
	IESR3_Avoidance	1.4 (2.1)	3.1 (3.3)	12
IESR6_Intrusion<br>IESR6_Hyperarousal<br>IESR6_Avoidance	Cluster distribution: Frequency (%)	22 (37.9)	31 (53.4)	--
	IESR3_Intrusion	0.5 (1.0)	1.9 (1.9)	20
	IESR3_Hyperarousal	0.1 (0.3)	1.5 (1.7)	27
	IESR3_Avoidance	1.3 (1.9)	2.3 (2.4)	7
IESR6_Numbing<br>IESR6_Irritability<br>IESR6_Sleep	Cluster distribution: Frequency (%)	22 (37.9)	32 (55.2)	--
	IESR3_Numbing	0.1 (0.5)	1.1 (1.6)	16
	IESR3_Irritability	0.04 (0.2)	0.6 (0.9)	17
	IESR3_Sleep	0.5 (0.6)	1.6 (1.6)	12
PSS_Avoidance<br>PSS_Arousal<br>PSS_Reexperiencing	Cluster distribution: Frequency (%)	23 (39.7)	33 (56.9)	--
	PSS_Avoidance	1.7 (2.0)	1.9 (1.9)	1
	PSS_Arousal	2.9 (2.9)	3.4 (2.6)	2
	PSS_Reexperiencing	0.8 (1.2)	1.8 (2.0)	9

Supplementary Table S6: Two-Step cluster model fit of trauma variables and hypertension

Input variables	Hypertension			
	**Clusters**	**AIC**	**Silhoutte**	**Ratio of sizes: largest to smallest cluster**
IES-R<br>PSS-SR	1	22.0		
	**2**	**20.8**	**0.51**	**3.23**
	3	30.0		
	4	40.0		
IESR3_Intrusion<br>IESR3_Hyperarousal<br>IESR3_Avoidance	1	30.4		
	**2**	**28.2**	**0.45**	**3.15**
	3	42.0		

Supplementary Table S7: Cluster characteristics of Two-Step cluster involving trauma variables and hypertension

Input variables	Criteria	Hypertention		Predictor importance (%)
		**Cluster 1** (Normal blood pressure)	**Cluster 2** (Hypertension)	
IES-R<br>PSS-SR	Cluster distribution: Frequency (%)	42 (72.4%)	13 (22.4%)	--
	IES-R	5.4 (6.4)	12.4 (10.7)	17
	PSS-SR	5.7 (5.0)	7.8 (5.4)	5
IESR3_Intrusion<br>IESR3_Hyperarousal<br>IESR3_Avoidance	Cluster distribution: Frequency (%)	41 (70.7)	13 (22.4)	--
	IESR3_Intrusion	1.7 (2.3)	4.5 (4.0)	21
	IESR3_Hyperarousal	1.4 (1.8)	3.3 (3.3)	15
	IESR3_Avoidance	1.7 (2.4)	4.5 (3.7)	21
